# Root Development Following Bioceramic Material Application in Immature Permanent Teeth: A Case Series With 24-Month Follow-Up

**DOI:** 10.1155/crid/1530438

**Published:** 2025-10-22

**Authors:** Yasser Alsayed Tolibah, Nada Bshara, Mohammad Tamer Abbara, Marwan Alhaji, Osama Aljabban, Ibrahim Ali Ahmad, Ziad D. Baghdadi

**Affiliations:** ^1^Department of Pediatric Dentistry, Damascus University, Damascus, Syria; ^2^Department of Endodontics, Damascus University, Damascus, Syria; ^3^Dentistry Department, Al-Wakra Hospital, Hamad Medical Corporation, Doha, Qatar; ^4^Division of Pediatric Dentistry, Department of Preventive Dental Sciences, University of Manitoba, Winnipeg, Manitoba, Canada

**Keywords:** apical barrier, bioceramics, immature teeth, periapical healing

## Abstract

**Objective:**

Dental trauma frequently impacts children and adolescents, leading to functional and esthetic challenges. If not managed promptly and effectively, complications may ensue. Apexification with calcium silicate cement (CSC) has been proposed as a reliable treatment for necrotic immature teeth with thick dentinal walls and open apices. While CSC effectively eliminates periapical infection and improves tooth survival, its ability to promote root lengthening in immature teeth is not consistently proven.

**Materials and Methods:**

This case series highlights five instances in four children of traumatized anterior teeth with necrotic pulps treated using bioceramic putty and bioceramic sealer—a contemporary generation of CSC—in apexification procedures spanning two to three visits.

**Results:**

Radiographic follow-ups of 24 months demonstrated successful canal system sealing, periapical lesions healing, and notable root lengthening. Importantly, no posttreatment complications, such as pain or exacerbation, were observed, underscoring the efficacy and potential of these materials for managing necrotic immature permanent teeth.

**Conclusion:**

Bioceramic apical barrier is recognized as an effective treatment for managing traumatized anterior teeth. Moreover, it demonstrates promising results by facilitating the healing of necrotic immature teeth and potentially supporting root lengthening.

**Trial Registration:**

ClinicalTrials.gov identifier: NCT06119477

## 1. Introduction

Dental trauma remains a prevalent issue among children and young adults, with an estimated 25% of school-aged children and 33% of adults experiencing trauma to permanent teeth [1]. The most common dental hard tissue injuries in permanent dentition are simple crown fractures followed by complicated ones [2]. While these injuries can generally be managed, immature permanent teeth are especially susceptible to complications if treatment is delayed or inadequate, leading to pulp necrosis [3]. Addressing these challenges in immature anterior teeth requires a nuanced approach that balances esthetics, function, and effective management of the root canal system [4]. For immature permanent teeth with open apices, the complex root morphology often precludes conventional endodontic treatment, necessitating specialized techniques such as apexification or regenerative endodontic therapy [5].

Apexification with calcium hydroxide dressings (CHDs) or the use of mineral trioxide aggregate (MTA) in the apical barrier technique has been the primary treatment option for immature nonvital permanent teeth [6].

Apical barrier technique with calcium silicate cement apical plugs has been indicated for necrotic immature permanent teeth treatment, particularly when root development aligns with Stages 2–4 of Cvek's classification [7].

The apical barrier technique involves the placement of a biocompatible material to create a 3–4 mm artificial barrier at the level of an open apex. This barrier is compacted and adapted using specialized carriers and pluggers to ensure a proper seal [8]. Following the establishment of the apical barrier, the remaining portion of the canal is filled either with gutta-percha, the same bioceramic material used for the apical plug, or restorative materials, particularly in cases with significant coronal tooth structure loss where additional reinforcement is required [9].

Recently, new generations of calcium silicate–based materials, such as Biodentine, BioAggregate, calcium-enriched mixture (CEM), bioactive glass, and bioceramics, have been introduced. These materials feature improved composition, enhanced handling properties, optimized viscosity, and faster setting times, offering significant benefits for clinical application in the apical barrier treatments [10].

According to the American Association of Endodontists (AAE), clinical and radiographic success of apical barrier treatments is defined by the resolution of clinical symptoms such as pain, swelling, and pathological mobility, as well as radiographic evidence of healing, including the disappearance of periapical lesions and the absence of pathological signs such as internal or external root resorption [11]. However, opinions are varied regarding its ability to promote root growth in immature teeth using apexification; a randomized controlled trial reported that apexification achieved with MTA apical plugs did not significantly contribute to root lengthening, whereas Biodentine apical plugs showed slight root elongation in a limited number of cases [12, 13]. Another investigation observed that MTA apical plugs resulted in minimal root length increases when compared to revascularization procedures after 1 year of follow-up [14]. In contrast, another study found no significant difference between apexification with MTA apical plugs and revascularization, as both approaches demonstrated comparable efficacy in promoting root length after a mean follow-up period of 19 months [15].

The current case series describes the management of five nonvital immature maxillary central incisors resulting from dental trauma, treated using the apical barrier technique with bioceramic putty and bioceramic sealer materials. This report provides detailed information on the clinical and radiographic outcomes associated with the use of these materials.

## 2. Materials and Methods

### 2.1. Subject Population

The study was conducted according to the Helsinki Declaration for Clinical Studies and approved by the Faculty of Dentistry Ethics Committee of Damascus University (UDDS-361-13032023/SRC-2654). The reporting of this case series followed the CAse REport (CARE) 2013 guidelines [16] and the Preferred Reporting Items for Case reports in Endodontics (PRICE) 2020 guidelines [17, 18]. This case series included five traumatized immature maxillary central incisors in four healthy pediatric patients who were referred to the Pediatric Dentistry Department at Damascus University's Faculty of Dentistry—the teeth presented with necrotic pulps and periapical lesions secondary to dental trauma caused by crown fractures. No abnormalities were observed during the extraoral examination. The demographic and clinical data of the included patients are summarized in Table 1.

Written informed consents were obtained from the patient's parents after explaining the apexification procedure, risks, and benefits.

### 2.2. Clinical Procedure

In all cases, preoperative periapical radiographs were taken using a digital dental x-ray unit (HyperLight; Eighteeth, Changzhou, China) and a digital sensor (#1.5 EzSensor Classic; Vatech, Gyeonggi-do, Korea) with the following settings: an exposure time of 0.2 s, 65 kVp, and 2.5 mA. All children were managed using basic behavior management techniques, including the Tell-Show-Do method and positive reinforcement, throughout all stages of treatment.

### 2.3. Canal Instrumentation, Debridement, and Disinfection Protocol

All carious tissue and prior restorations were removed after infiltration anesthesia (Huons Lidocaine HCL, Seoul, Korea), and rubber dam isolation (Sanctuary, Perak, Malaysia) was administered. Rubber dam isolation in Case 1 was achieved from the first premolar to the contralateral first premolar, with clamps secured on the maxillary premolars. In the remaining cases, isolation was performed on a single tooth using a clamp placed on the targeted tooth. Access cavities were refined with an Endo-Z bur (Dentsply Maillefer, Tulsa, Oklahoma, United States), and canal orifices were prepared gently with an orifice opener file (Orodeka Ltd., Xincheng, Jining, China).

The working length was determined radiographically using an appropriately sized K-file set 1 mm short of the radiographic apex. Root canals were gently shaped and debrided using 25/06 Plex V ORODEKA rotary files (Orodeka Ltd., Xincheng, Jining, China). After instrumentation, each canal was filled with NaOCl, irrigated with 10 mL of 5.25% NaOCl, dried with sterile paper points (Gabadent, Guangdong, China), and filled with a CHD. Then, each tooth was temporized with glass ionomer filling (Medifil; Promedica Dental Material GmbH, Neumunster, Germany).

After 14 days, under local anesthesia and isolation, the temporary filling and CHD were removed. A final irrigation was conducted by filling each canal with 5.25% NaOCl and activating it with an XP-endo Finisher file (FKG Dentaire, La Chaux-de-Fonds, Switzerland) for 30 s at 800 rpm and 1 N·cm, covering the entire canal wall to remove remaining necrotic pulp and CHD debris. This activation was repeated with 10 mL of NaOCl over 5 min. Following irrigation with 5 mL of saline and 2 mL of 17% EDTA (Dentsply Tulsa Dental, Tulsa, Oklahoma, United States), activated with the XP-endo Finisher file for 15 s (twice), canals were rinsed again with 5 mL of saline, 5 mL of 5.25% NaOCl, and 5 mL of saline.

### 2.4. Root Canal System Obturation

#### 2.4.1. Case 1 (Figure 1)

In Tooth #21, a bioceramic putty (TotalFill BC RRM, FKG Dentaire, Le Crêt-du-Locle, Switzerland) was applied to the apical 4 mm of the canal using a modified cannula [8], with placement adapted using a hand plugger and confirmed radiographically. A minor extrusion of the bioceramic putty beyond the apex was noted during obturation. Then, the tooth was temporized with a cotton pellet and glass ionomer filling. The following day, after isolation and removal of the temporary filling and cotton pellet, the remaining canal space was obturated with gutta-percha and bioceramic sealer (TotalFill BC Sealer, FKG Dentaire, Le Crêt-du-Locle, Switzerland) using the cold lateral condensation technique. A periapical radiograph confirmed that the canal was obturated without voids or gaps.

#### 2.4.2. Case 2 (Figure 2)

In Tooth #21, the whole root canal was filled using a 90/0.02 master gutta-percha cone (DiaDent, Cheongju, Korea) and TotalFill BC Sealer. The postoperative periapical radiograph confirmed that the canal was obturated without voids or gaps and without extrusion of obturation materials.

#### 2.4.3. Case 3 (Figure 3)

In Tooth #21, the canal was initially filled with a bioceramic sealer (TotalFill BC Sealer), followed by the insertion of four small pellets of bioceramic putty (TotalFill BC RRM) at the canal orifice, which was then gently compacted using hand pluggers. A periapical radiograph confirmed that the canal was obturated without voids or gaps. A minor extrusion of the bioceramic sealer beyond the apex was noted during obturation.

Bioceramic sealer beyond the apex was noted during obturation.

#### 2.4.4. Case 4 (Figure 4)

In Tooth #11, TotalFill BC RRM bioceramic putty was applied to the apical 4 mm of the canal using a modified cannula, with placement adapted using a hand plugger and confirmed radiographically. Then, the tooth was temporized with a cotton pellet and glass ionomer filling. The following day, after isolation and removal of the temporary filling and cotton pellet, the remaining canal space was obturated with gutta-percha and TotalFill BC Sealer bioceramic sealer using the cold lateral condensation technique. A periapical radiograph confirmed that the canal was obturated without voids or gaps.

In Tooth #21, the whole root canal was filled using a 120/0.02 master gutta-percha cone and TotalFill BC Sealer. The postoperative periapical radiograph confirmed that the canal was obturated without voids or gaps and without extrusion of filling materials.

### 2.5. Coronal Restoration

The access cavities of all teeth were sealed with composite restorations (Tetric N-Ceram; Lichtenstein, Ivoclar Vivadent, Leicester, United Kingdom) on the same day of filling the coronal portion of the canal. However, the final restoration of Tooth 21 in Case #4 was postponed after filling the canal and was performed in the same session as the restoration of Tooth 11. This decision was made to ensure symmetry in the restorations and to facilitate the treatment process. During periodic follow-up appointments, finishing and polishing of the final restorations were performed as needed to maintain optimal surface integrity and esthetics.

## 3. Results

Follow-up radiographs were exposed at 6, 12, and 24 months postoperatively using the same exposure settings as the preoperative radiographs. Sensor holders (Dentsply Sirona Inc., Charlotte, North Carolina, United States) were utilized to ensure consistent imaging angles, improving the accuracy and reliability of radiographic comparisons over time.

All cases demonstrated root lengthening compared to the adjacent healthy teeth, with calcified tissue formation observed apically to the materials used for apical sealing. Additionally, a comparison between the apex shape in the initial diagnostic and the follow-up radiograph after 12 months revealed noticeable changes, with calcified tissue formation observed apically to the materials used for apical sealing. Moreover, a healthy periodontal space was also noted, along with the resolution of the periapical lesion and intact bone formation.

In the first and third cases, no resorption of the extruded material was observed, and the surrounding bone was intact after 12 months of the treatment. In contrast, in the first case, partial resorption of the extruded material was observed after 24 months of follow-up. However, complete resolution was not achieved, although the surrounding alveolar bone remained intact and stable.

In the second case, after 12 months of follow-up, the immature incisor demonstrated a slight increase in root length compared to the other cases, highlighting the variability in the periapical cellular response to these materials among individuals over the same period. However, after 24 months of follow-up, this case also demonstrated additional root lengthening similar to the other cases in the series. This finding suggests that root development may have continued despite initial radiographic evidence of apical closure, highlighting the dynamic nature of healing and maturation following bioceramic treatment.

Notably, no posttreatment flare-up symptoms occurred, and all cases demonstrated complete clinical success at the 6-, 12-, and 24-month follow-ups.

**Figure 5:**
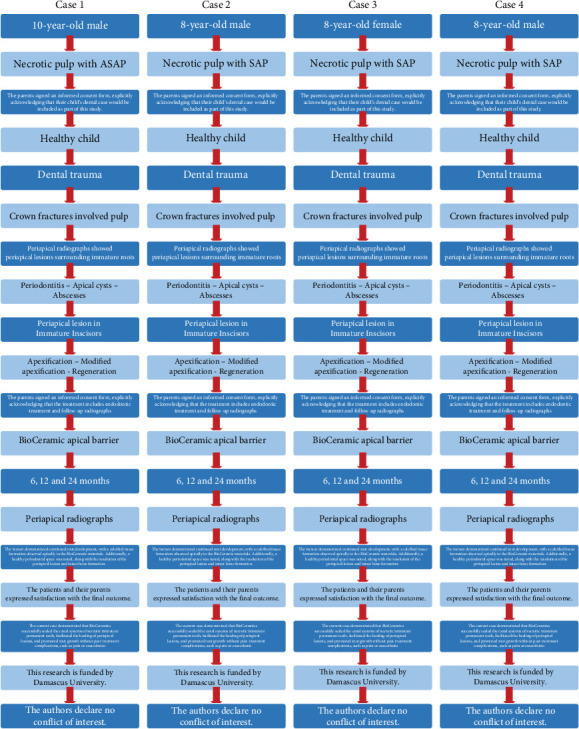
PRICE 2020 flowchart showing the steps involved in the case series.

Figure 5 summarizes the diagnostic and treatment approaches for this case series.

## 4. Discussion

With advancements in dental materials over recent decades, understanding their effects on necrotic immature teeth healing has become essential for optimizing the management of dental trauma and improving clinical outcomes and prognoses [19].

Bioceramics are increasingly utilized as apical barrier materials due to their excellent biocompatibility, slight expansion upon setting, strong chemical bonding with dentin via hydroxyapatite formation, superior sealing ability in moist environments, and inherent antibacterial pH—making them particularly well suited for achieving reliable apical closure in apexification procedures [10, 20–23].

In a previous case report [24], periapical tissue regeneration of approximately 2 mm was observed following MTA plug placement in a case monitored for 2 years, while another case observed over 5 years showed a radicular length increase exceeding 2 mm. Based on these two cases and the studies discussed, it is speculated that the MTA apical plug, when placed in contact with the apical papilla, may have stimulated dental tissue regeneration—promoting radicular elongation and apical closure—rather than merely serving as a hard tissue barrier. Moreover, a previous case report [13] documented the successful apexification of a nonvital immature mandibular second premolar using EndoSequence Root Repair Material (ERRM) bioceramic material (BC RRM-Fast Set Putty, Brasseler USA). The 6-month follow-up showed significant periapical healing with the continuation of the root growth to achieve its “matured development.” The previous cases sparked interest in using calcium silicate cement materials for apexification. However, both reports concluded that more evidence and studies are needed to verify this outcome.

The use of bioceramics in the presented cases has been investigated through three distinct techniques. For instance, Sockalingam et al. [13] recommended the application of bioceramic putty as an apical plug. Rencher et al. [25] demonstrated that bioceramic putty and bioceramic sealer were employed for retrograde filling after apicoectomy. Additionally, bioceramic sealer use in conjunction with the single-cone technique was based on manufacturer guidelines, with reports of high success rates in managing large periapical lesions in closed-apex anterior teeth [26]. However, this technique requires modifications for open-apex cases, as the gutta-percha cone cannot achieve tug-back at the open apex. Instead, tug-back is accomplished at the apical third of the root, where the bioceramic sealer's high flowability seals the gap between the gutta-percha cone and apical walls.

These presented cases achieved the clinical and radiographical criteria of success by the AAE guideline [11]. Although the literature contains only a few reports of increased root length in immature teeth treated with the apical barrier technique alone, without additional regenerative interventions such as scaffolds or blood clot induction within the canal space [12–15], no established criterion for the success of apexification includes increased root length as a requirement. It is worth mentioning that the AAE considers increasing the root length a secondary, rather than primary, goal of regenerative endodontic procedures [11].

Apical bleeding induced during regenerative procedures provides a three-dimensional fibrin scaffold enriched with platelets and growth factors such as VEGF and TGF-*β*, which not only recruit stem cells of the apical papilla (SCAPs) but also stimulate their differentiation into odontoblast-like cells capable of depositing new dentin-like tissue, thereby contributing to continued root elongation and wall thickening. Moreover, the preservation of Hertwig's epithelial root sheath (HERS) is considered pivotal in regenerative endodontics, as its signaling regulates root morphogenesis; interaction between SCAP- and HERS-derived factors orchestrates apical matrix deposition and extension of the root in an immature tooth undergoing revascularization [27, 28].

The results observed in this case series can be attributed to the biological properties of bioceramic materials and their direct impact on stem cells in the periapical region. These properties offer a plausible explanation for periapical healing and root length increase. Research has demonstrated that calcium silicate–based biomaterials are biocompatible with human periodontal ligament stem cells, enhancing tube stimulation and promoting angiogenesis, ultimately supporting tissue regeneration [29].

Moreover, bioceramic sealers do not interfere with macrophage adhesion (MBL6 and MBalb) or compromise their ability to perform phagocytosis and produce transforming growth factor-beta. They also stimulate the production of reactive oxygen species in MBL6 macrophages, which play a role in combating residual pathogens during inflammatory responses [30]. Moreover, bioceramic sealers and bioceramic putty elicit specific immune responses, influencing the morphology and activity of CD14+ monocytes, suggesting a unique role for these materials in promoting the healing and regeneration of periapical lesions [31]. Additionally, bioceramic materials enhanced the osteogenic differentiation of MG-63 cells in vitro by increasing the expression of genes such as IL-1A and IL-6, suggesting their potential role in remineralizing bone lesions caused by pulp necrosis [32]. These findings align with the observed complete clinical resolution of periapical lesions following 12 months of monitoring.

SCAPs are resilient during pulp necrosis and the development of periapical lesions due to their strategic location near the neurovascular bundle, low metabolic requirements in a dormant state, and the rich vascularization of the apical region. These characteristics enable successful regenerative endodontic procedures even in cases involving periapical lesions [33]. In vitro studies further revealed that bioceramic putty increased SCAP viability by 121%, ensuring survival and promoting cellular proliferation and growth. This material upregulates ALP gene expression, which is critical for mineralization and activating osteoblasts and odontoblasts. It also elevates RNA expression of dentin sialophosphoprotein, a marker for odontoblast differentiation. These findings suggest that bioceramic putty facilitates SCAP differentiation into odontoblast-like cells, which may account for the formation of dentin-like tissue observed at the interface between the SCAP/periapical lesion region and the bioceramic surface [34].

In addition to the aforementioned favorable properties of bioceramics, it is important to highlight the role of EDTA in the final irrigation protocol described in previous cases. EDTA irrigation facilitates the release of dentin-derived growth factors, and the subsequent ionic exchange at the canal wall creates a microenvironment conducive to mineralized tissue formation [35]. Together, these processes enhance apical closure and may explain the radiographic evidence of root lengthening observed after revascularization therapy.

The presented case series highlights the potential use of bioceramic putty and bioceramic sealers in achieving apical closure while promoting root lengthening beneath the apical plug. This approach circumvents the complexity of regenerative procedures, which carry variable success rates [5]. By leveraging this method, the remaining canal space can be utilized for coronal restoration, particularly in trauma cases involving significant loss of coronal structure. In the presented cases, bioceramic apexification partially resembled the regenerative treatments by eliminating periapical pathologies and encouraging the formation of new tissues. However, bioceramics did not facilitate intertissued growth within the canal system, which would be necessary for outcomes such as increased root thickness, the formation of pulp-like tissues, or the restoration of tooth sensitivity. This distinction underscores that bioceramics contribute significantly to healing and structural support. However, their regenerative capacity within the canal system is limited compared to the complex cellular and molecular dynamics enabled by accurate biological scaffolds in regenerative endodontics.

The extrusion of bioceramic materials during endodontic treatment is a known possibility due to their high flowability, with studies reporting an occurrence rate of approximately 47.4% in closed apical canals. This likelihood naturally increases in cases with open apices. Moreover, the bioceramic extrusion did not affect treatment outcomes [36].

Consistent with previous retrospective studies [36, 37], complete resorption of extruded material at the apex was not observed. In the first case, the extruded material migrated away from the apex, coinciding with enhanced root formation around the apical area. Conversely, in the third case, the extruded material seemed to integrate into the newly formed tissue, contributing to root elongation. However, definitive conclusions regarding the extruded material's role in promoting root development cannot be drawn, as the second and fourth cases, which did not involve material extrusion, also demonstrated root formation. Further radiographic studies are essential to establish a distinct relationship between bioceramic material extrusion and root development.

The described techniques were part of a research study to identify the most effective bioceramic apexification method for managing traumatized immature necrotic teeth in children. Our next step is to evaluate the differences between these techniques in treatment duration, posttreatment pain, extrusion of obturation materials, discoloration, periapical lesion healing, and tooth survival rates. Such research will help identify the optimal approach for managing dental trauma in children and achieving the best possible prognosis.

Some limitations were noted. Advanced imaging, such as micro–computed tomography, could provide a three-dimensional understanding of the newly calcified formed tissue density and spatial arrangement. However, its routine use in pediatric patients may not be warranted. Further research, including animal studies and histological evaluations, is necessary to define the exact nature of the regenerated tissues. Additionally, randomized controlled trials are needed to compare bioceramic materials with the standard MTA in managing necrotic immature teeth with varying periapical lesion sizes.

## 5. Conclusions

The current cases demonstrated that bioceramic materials successfully sealed the canal systems of necrotic immature permanent teeth, facilitated the healing of periapical lesions, and promoted root growth without posttreatment complications, such as pain or exacerbation. However, further histological, clinical, and randomized controlled radiographic studies are necessary to deepen the understanding of apical barrier treatments' bioceramic behavior and confirm their long-term efficacy and outcomes.

## Figures and Tables

**Figure 1:**
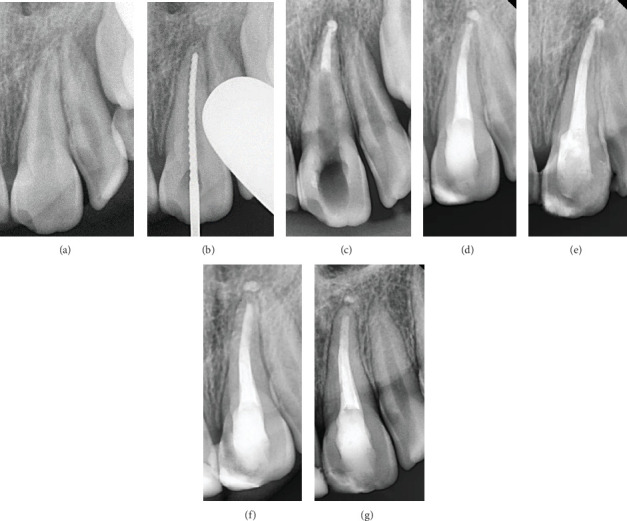
Case 1. (a) Preoperative periapical radiograph. (b) Working length radiograph. (c) Intermediate radiograph showing apical plug formation. (d) Postoperative radiograph. (e) A 6-month recall radiograph. (f) A 12-month recall radiograph. (g) A 24-month recall radiograph.

**Figure 2:**
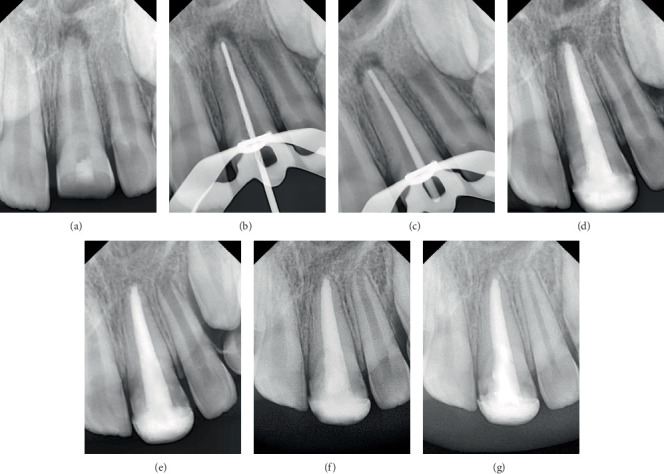
Case 2. (a) Preoperative periapical radiograph. (b) Working length radiograph. (c) Cone fit radiograph. (d) Postoperative radiograph. (e) A 6-month recall radiograph. (f) A 12-month recall radiograph. (g) A 24-month recall radiograph.

**Figure 3:**
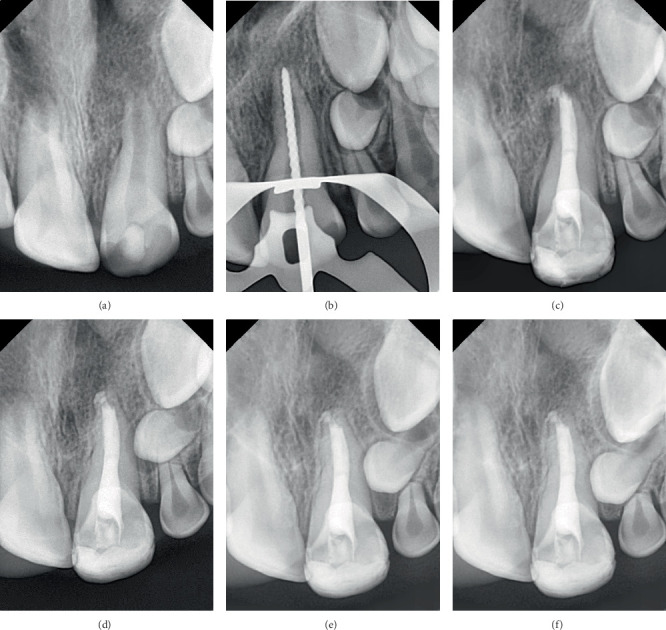
Case 3. (a) Preoperative periapical radiograph. (b) Working length radiograph. (c) Postoperative radiograph. (d) A 6-month recall radiograph. (e) A 12-month recall radiograph. (f) A 24-month recall radiograph.

**Figure 4:**
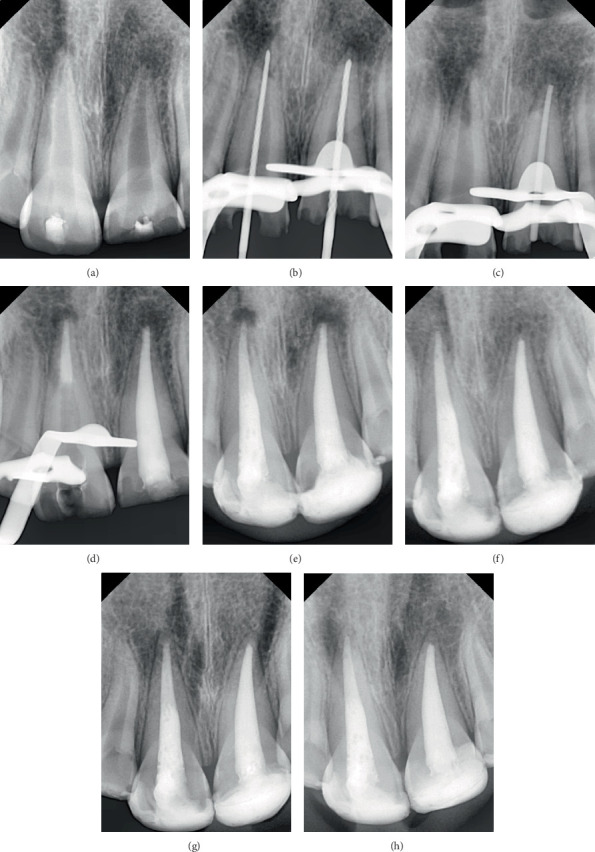
Case 4. (a) Preoperative periapical radiograph. (b) Working length radiograph. (c) Cone fit radiograph (Tooth #21). (d) Intermediate radiograph showing apical plug formation in Tooth #11 and obturation of Tooth #21. (e) Postoperative radiograph. (f) A 6-month recall radiograph. (g) A 12-month recall radiograph. (h) A 24-month recall radiograph.

**Table 1:** The demographic and clinical data of included patients.

**Case**	**Gender**	**Age (years)**	**Ethnicity**	**Tooth**	**Diagnosis**	**Etiology of pulp necrosis**	**Apical size (mm)**	**Filling method**	**Bioceramic extrusion**
1	Male	10	Middle Eastern	21	Necrotic pulp with ASAP	Complicated crown fracture	1.0	BC putty apical plug and coronal CLC (GP and BC sealer)	Yes
2	Male	8	Middle Eastern	21	Necrotic pulp with SAP	Complicated crown fracture	1.1	SCGP and BC sealer	No
3	Female	8	Middle Eastern	21	Necrotic pulp with ASAP	Complicated crown fracture	1.5 >	BC putty and sealer	Yes
4	Male	8	Middle Eastern	11	Necrotic pulp with ASAP	Complicated crown fracture	0.9	BC putty apical plug and coronal CLC (GP and BC sealer)	No
				21	Necrotic pulp with ASAP	Complicated crown fracture	1.2	SCGP and BC sealer	No

Abbreviations: ASAP, asymptomatic apical periodontitis; BC, bioceramic; CLC, cold lateral condensation; GP, gutta-percha; SAP, symptomatic apical periodontitis; SCGP, single-cone gutta-percha.

## Data Availability

Data are available on request from the authors.

## References

[1] Levin L., Day P. F., Hicks L. (2020). International Association of Dental Traumatology Guidelines for the Management of Traumatic Dental Injuries: General Introduction. *Dental Traumatology*.

[2] Choi S. C., Park J. H., Pae A., Kim J. R. (2010). Retrospective Study on Traumatic Dental Injuries in Preschool Children at Kyung Hee Dental Hospital, Seoul, South Korea. *Dental Traumatology*.

[3] Reddy L. V., Bhattacharjee R., Misch E., Sokoya M., Ducic Y. (2019). Dental Injuries and Management. *Facial Plastic Surgery*.

[4] Chotvorrarak K., Danwittayakorn S., Banomyong D., Suksaphar W. (2024). Intraradicular Reinforcement of Traumatized Immature Anterior Teeth After MTA Apexification. *Dental Traumatology*.

[5] Murray P. E. (2023). Review of Guidance for the Selection of Regenerative Endodontics, Apexogenesis, Apexification, Pulpotomy, and Other Endodontic Treatments for Immature Permanent Teeth. *International Endodontic Journal*.

[6] Chugal N., Mallya S. M., Kahler B., Lin L. M. (2017). Endodontic Treatment Outcomes. *Dental Clinics of North America*.

[7] Duncan H. F., Kirkevang L. L., Peters O. A. (2023). Treatment of Pulpal and Apical Disease: The European Society of Endodontology (ESE) S3-Level Clinical Practice Guideline. *International Endodontic Journal*.

[8] Tolibah Y. A., Droubi L., Alkurdi S. (2022). Evaluation of a Novel Tool for Apical Plug Formation During Apexification of Immature Teeth. *International Journal of Environmental Research and Public Health*.

[9] Crozet A., Aubeux D., Pérez F., Gaudin A. (2023). Fracture Resistance of Simulated Immature Maxillary Anterior Teeth Restored With Various Canal Filling Materials, With Micro-Posts or With a Fiber Post. *Dental Materials Journal*.

[10] Dong X., Xu X. (2023). Bioceramics in Endodontics: Updates and Future Perspectives. *Bioengineering*.

[11] American Association of Endodontists (2016). *AAE Clinical Considerations for a Regenerative Procedure*.

[12] Tolibah Y. A., Kouchaji C., Lazkani T., Ahmad I. A., Baghdadi Z. D. (2022). Comparison of MTA Versus Biodentine in Apexification Procedure for Nonvital Immature First Permanent Molars: A Randomized Clinical Trial. *Children*.

[13] Sockalingam S., Awang Talip M., Zakaria A. S. I. (2018). Maturogenesis of an Immature Dens Evaginatus Nonvital Premolar With an Apically Placed Bioceramic Material (EndoSequence Root Repair Material): An Unexpected Finding. *Case Reports in Dentistry*.

[14] Lin J., Zeng Q., Wei X. (2017). Regenerative Endodontics Versus Apexification in Immature Permanent Teeth With Apical Periodontitis: A Prospective Randomized Controlled Study. *Journal of Endodontics*.

[15] Pereira A. C., Oliveira M. L., Cerqueira-Neto A. C. C. L. (2021). Outcomes of Traumatised Immature Teeth Treated With Apexification or Regenerative Endodontic Procedure: A Retrospective Study. *Australian Endodontic Journal*.

[16] Gagnier J. J., Kienle G., Altman D. G. (2013). The CARE Guidelines: Consensus-Based Clinical Case Reporting Guideline Development. *BML Case Reports*.

[17] Nagendrababu V., Chong B. S., McCabe P. (2020). PRICE 2020 Guidelines for Reporting Case Reports in Endodontics: A Consensus-Based Development. *International Endodontic Journal*.

[18] Nagendrababu V., Chong B. S., McCabe P. (2020). PRICE 2020 Guidelines for Reporting Case Reports in Endodontics: Explanation and Elaboration. *International Endodontic Journal*.

[19] Gladwin L., Darcey J. (2023). The Consequences of Dental Trauma. *Primary Dental Journal*.

[20] Ghaly M. S., Abozena N. I., Ghouraba R. F., Kabbash I. A., el-Desouky S. S. (2025). Clinical and Radiographic Evaluation of Premixed Bioceramic Putty as an Apical Plug in Nonvital Immature Anterior Permanent Teeth. *Scientific Reports*.

[21] López-García S., Pecci-Lloret M. R., Guerrero-Gironés J. (2019). Comparative Cytocompatibility and Mineralization Potential of Bio-C Sealer and TotalFill BC Sealer. *Materials*.

[22] Wang Z., Zhang J., Sun X. (2024). Nanoparticulate Bioceramic Putty Suppresses Osteoclastogenesis and Inflammatory Bone Loss in Mice via Inhibition of TRAF6-Mediated Signalling Pathways: A Laboratory Investigation. *International Endodontic Journal*.

[23] Knapp J., Kirkpatrick T., Ontiveros J. C., Jaramillo D. E., Kim H. C., Jeong J. W. (2024). Efficacy of Root-End Filling Techniques Using Premixed Putty Type Bioceramic Cements: An Ex Vivo Study. *Clinical Oral Investigations*.

[24] Masmoudi F., Bourmeche I., Sebai A., Baccouche Z., Maatouk F. (2018). Root Lengthening With Apical Closure in Two Maxillary Immature Permanent Central Incisors After Placement of Mineral Trioxide Aggregate (MTA) as an Apical Plug. *European Archives of Paediatric Dentistry*.

[25] Rencher B., Chang A. M., Fong H., Johnson J. D., Paranjpe A. (2021). Comparison of the Sealing Ability of Various Bioceramic Materials for Endodontic Surgery. *Restorative Dentistry & Endodontics*.

[26] Wahbi E., Achour H., Alsayed Tolibah Y. (2024). Comparison Between AH Plus Sealer and Total Fill Bioceramic Sealer Performance in Previously Untreated and Retreatment Cases of Maxillary Incisors With Large-Sized Periapical Lesion: A Randomized Controlled Trial. *BDJ Open*.

[27] Shahoon H., Dehghani Soltani A., Dehghani Soltani H., Salmani Z., Sabzevari B., Sajedi S. M. (2025). Clinical Outcomes of Biomaterial Scaffolds in Regenerative Endodontic Therapy: A Systematic Review and Meta-Analysis. *European Endodontic Journal*.

[28] Alothman F. A., Hakami L. S., Alnasser A., AlGhamdi F., Alamri A. A., Almutairii B. M. (2024). Recent Advances in Regenerative Endodontics: A Review of Current Techniques and Future Directions. *Cureus*.

[29] Olcay K., Taşli P. N., Güven E. P. (2020). Effect of a Novel Bioceramic Root Canal Sealer on the Angiogenesis-Enhancing Potential of Assorted Human Odontogenic Stem Cells Compared With Principal Tricalcium Silicate-Based Cements. *Journal of Applied Oral Science*.

[30] Tavares L. C. T., Canal Vasconcellos B. . L., Maia C. A. (2024). The Influence of Bioceramic Cements on the Activity of Macrophages of Different Lineages: A Laboratory Study. *Journal of Endodontics*.

[31] Castro-Jara S., Antilef B., Osbén C. (2023). Bioactivity Analysis of Calcium Silicate-Based Sealers and Repair Cements on the Phenotype and Cytokine Secretion Profile of CD14^(+)^ Monocytes: An Ex Vivo Study. *International Endodontic Journal*.

[32] Pasqualini D., Comba A., Annaratone L. (2020). Osteogenic Potential of Fast Set Bioceramic Cements: Molecular and In Vitro Study. *Applied Sciences*.

[33] Liu Q., Gao Y., He J. (2023). Stem Cells From the Apical Papilla (SCAPs): Past, Present, Prospects, and Challenges. *Biomedicine*.

[34] Miller A. A., Takimoto K., Wealleans J., Diogenes A. (2018). Effect of 3 Bioceramic Materials on Stem Cells of the Apical Papilla Proliferation and Differentiation Using a Dentin Disk Model. *Journal of Endodontics*.

[35] Galler K. M. (2016). Clinical Procedures for Revitalization: Current Knowledge and Considerations. *International Endodontic Journal*.

[36] Chybowski E. A., Glickman G. N., Patel Y., Fleury A., Solomon E., He J. (2018). Clinical Outcome of Non-Surgical Root Canal Treatment Using a Single-Cone Technique With Endosequence Bioceramic Sealer: A Retrospective Analysis. *Journal of Endodontics*.

[37] Li J., Chen L., Zeng C., Liu Y., Gong Q., Jiang H. (2022). Clinical Outcome of Bioceramic Sealer iRoot SP Extrusion in Root Canal Treatment: A Retrospective Analysis. *Head & Face Medicine*.

